# Bi- and trinuclear copper(I) complexes of 1,2,3-triazole-tethered NHC ligands: synthesis, structure, and catalytic properties

**DOI:** 10.3762/bjoc.12.85

**Published:** 2016-05-03

**Authors:** Shaojin Gu, Jiehao Du, Jingjing Huang, Huan Xia, Ling Yang, Weilin Xu, Chunxin Lu

**Affiliations:** 1 School of Materials Science and Engineering, Wuhan Textile University, Wuhan 430200, People's Republic of China; 2 College of Biological, Chemical Sciences and Engineering, Jiaxing University, Jiaxing 314001, People's Republic of China

**Keywords:** copper, CuAAC reaction, : N-heterocylic carbene, 1,2,3-triazole

## Abstract

A series of copper complexes (**3**–**6**) stabilized by 1,2,3-triazole-tethered N-heterocyclic carbene ligands have been prepared via simple reaction of imidazolium salts with copper powder in good yields. The structures of bi- and trinuclear copper complexes were fully characterized by NMR, elemental analysis (EA), and X-ray crystallography. In particular, [Cu_2_(L_2_)_2_](PF_6_)_2_ (**3**) and [Cu_2_(L_3_)_2_](PF_6_)_2_ (**4**) were dinuclear copper complexes. Complexes [Cu_3_(L_4_)_2_](PF_6_)_3_ (**5**) and [Cu_3_(L_5_)_2_](PF_6_)_3_ (**6**) consist of a triangular Cu_3_ core. These structures vary depending on the imidazolium backbone and N substituents. The copper–NHC complexes tested are highly active for the Cu-catalyzed azide–alkyne cycloaddition (CuAAC) reaction in an air atmosphere at room temperature in a CH_3_CN solution. Complex **4** is the most efficient catalyst among these polynuclear complexes in an air atmosphere at room temperature.

## Introduction

N-Heterocyclic carbene (NHC) have interesting electronic and structural properties. This resulted in their use as versatile ligands in organometallic chemistry and homogeneous catalysis [1–12]. A number of transition metal complexes of NHCs containing pyridine [13], pyrimidine [14], pyrazole [15–16], naphthyridine [17], pyridazine [18], and phenanthroline [19–20] donating groups have been studied in metal-catalyzed organic transformations. Recently, the easy synthesis and versatile coordination ability of 1,2,3-triazoles have led to an explosion of interest in coordination chemistry [21] and homogeneous catalysis [22–26]. Although a number of metal complexes containing 1,4-disubstituted-1,2,3-triazole ligands were well studied, reports concerning their preparation and use of 1,4-disubstituted-1,2,3-triazoles bearing NHC ligands are rare [22–23]. Elsevier et al. [23] reported several of palladium(II) complexes containing a heterobidentate N-heterocyclic carbene-triazolyl ligand. These palladium(II) complexes are active precatalysts in the transfer semihydrogenation of alkynes to *Z*-alkenes. Messerle et al. [26] synthesized a series of new cationic Rh(I), Rh(III) and Ir(III) complexes containing hybrid bidentate N-heterocyclic carbene-1,2,3-triazolyl donors. We [27] have synthesized a series of nonsymmetrical pincer palladium and platinum complexes containing 1,2,3-triazole-tethered NHC ligands. The obtained palladium complexes displayed high activity in aqueous Suzuki–Miyaura cross-coupling reactions.

We are interested in the synthesis and use of functionalized NHC ligands [20,28–31]. Herein, the synthesis, structural characterization, and catalytic properties of a few copper-1,2,3-triazole-tethered NHC complexes is reported.

## Results and Discussion

### Synthesis and spectroscopic characterization

**Scheme 1:**
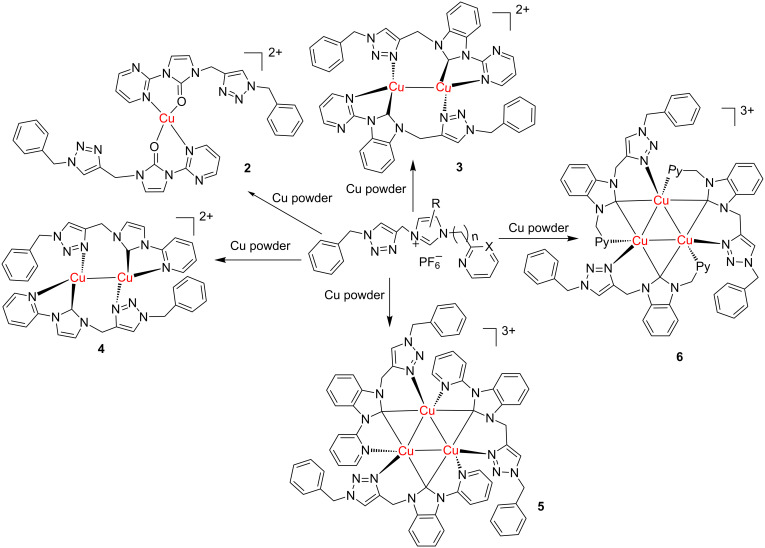
Synthesis of copper complexes **2–6**.

The imidazolium salts (**1a–e**) were prepared according to the reported procedure in 61–90% yields [27]. These imidazolium salts have been characterized by NMR spectroscopy. The ^1^H NMR spectra of these imidazolium salts show singlet peaks between 10.04 and 10.89 ppm in DMSO-*d*_6_. As seen in Scheme 1, copper–NHC complexes **3**–**6** can be obtained in 52–90% yields via directly reacting the corresponding imidazolium salts with an excess of copper powder in CH_3_CN at 50 °C for 5 h.

As shown in Scheme 1, reactions of the pyrimidine imidazolium salt **1a** with copper powder in acetonitrile afforded a light yellow Cu(II) complex. In complex **2**, the carbenic carbon atom was oxidized into carbonyl, which is similar with the reported pyrimidyl-imidazole complex [32]. However, a red binuclear Cu(I) complex **3** was obtained in 57% yield when we reacted pyrimidyl benzimidazolium salt **1b** with copper powder. Furtherly, we got a yellow Cu(I)–NHC complex **4** in about 70% yield from pyridine imidazolium salt **1c** and copper powder (Scheme 1). In addition, a triangular Cu(I) complex **6** can be obtained when a flexible ligand was used. Complex **6** consists of a triangular Cu_3_ core bridged by three NHCs, which is similar with the published Cu_3_ complexes containing flexible ligands [33]. Interestingly, we can also obtain a similar triangular Cu_3_ complex **5** rather than a binuclear copper complex using a rigid pyridine benzimidazolium salt **1d**. These results demonstrated that the structures vary depending on the N substituents and on the imidazolium backbone. Fine adjustment of the structure of the ligand can lead to different structures.

All of the prepared copper–NHC complexes are stable in air. They were fully characterized by NMR, elemental analysis (EA), and X-ray crystallography. The generation of these copper–NHC complexes were confirmed by the absence of the ^1^H NMR resonance signal of the acidic imidazolium protons between 10.04 and 10.89 ppm. The ^1^H NMR spectra of all the complexes display only one set of resonance signals assignable to the corresponding ligands, indicating two or three magnetically equivalent ligands. ^13^C NMR spectra of the copper(I) complexes showed their carbenic carbon resonances at 177.6–191.2 ppm, which are in the normal range of 157.6–216 ppm [34–35].

### Single crystal X-ray diffraction studies

To obtain additional insight into the coordination and supramolecular properties, suitable single crystals of all the copper complexes were obtained for single-crystal X-ray diffraction analysis. Crystals were grown by slow diffusion of diethyl ether into an acetonitrile solution of the copper complex at room temperature.

**Figure 1:**
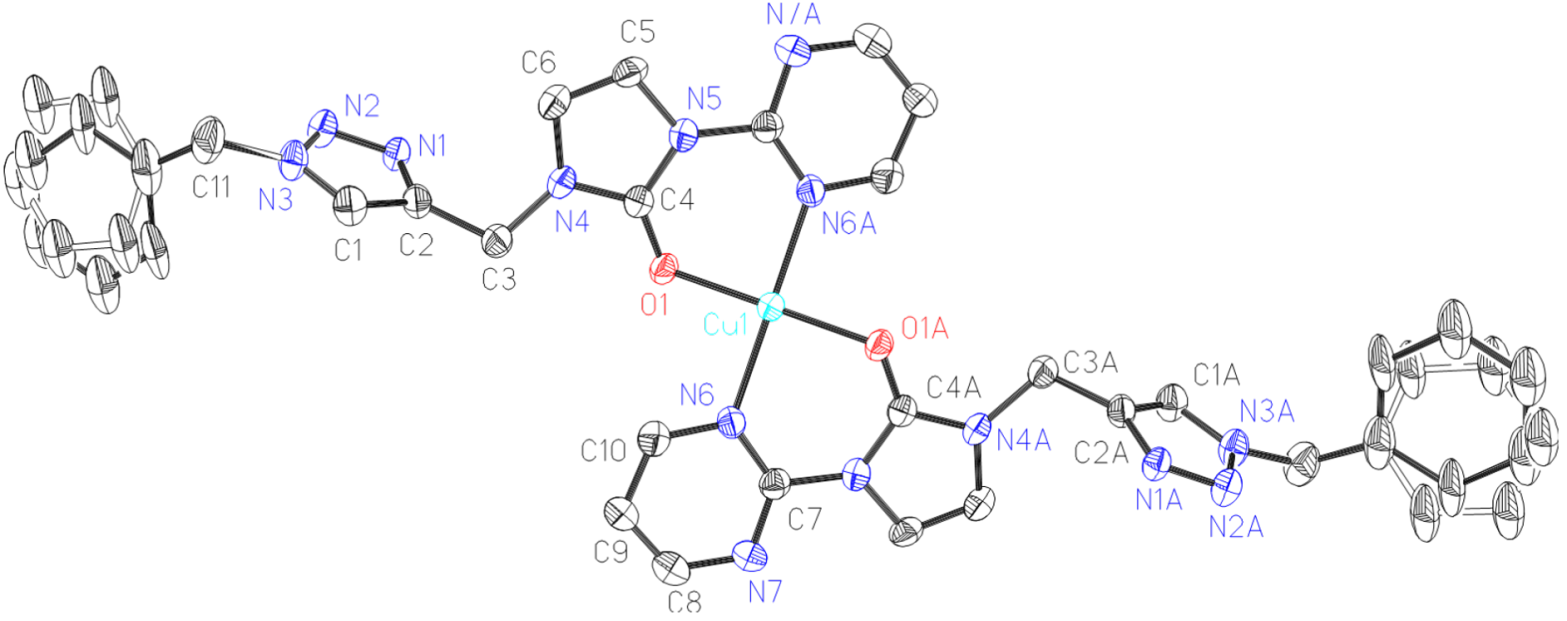
X-ray diffraction structure of copper(II) complex **2** with thermal ellipsoids drawn at 30% probability. The anion and hydrogen atoms are omitted for clarity. Selected bond distances (Å) and angles (°): Cu1-O1 1.931(4), Cu1-N6 2.042(5); O1-Cu1-O1A 180.0(3), O1-Cu1-N6A 90.5(2), O1-Cu1-N6 89.5(2), N6-Cu1-N6A 180.00(8). Symmetry transformations used to generate equivalent atoms: −X, Y, 0.5−Z.

Green-yellow single crystals of complex **2** suitable for an X-ray diffraction study were grown from acetonitrile solution and diethyl ether. The molecular structure of complex **2** in the solid state is depicted in Figure 1 along with the principal bond lengths and angles. Complex **2** crystallizes in the orthorhombic space group *Pnna*. The remaining atoms of the cation are related by a crystallographic 2-fold symmetry. In complex **2**, the copper ion is four-coordinate in a distorted square planar ligand environment of two nitrogen atoms and two oxyen atoms. The Cu–O bonds are in *trans* configuration and Cu–O distances are shorter than Cu–N distances. The two ligands are arranged in head-to-tail manner. And the N_triazole_ did not participate in the corrdination.

**Figure 2:**
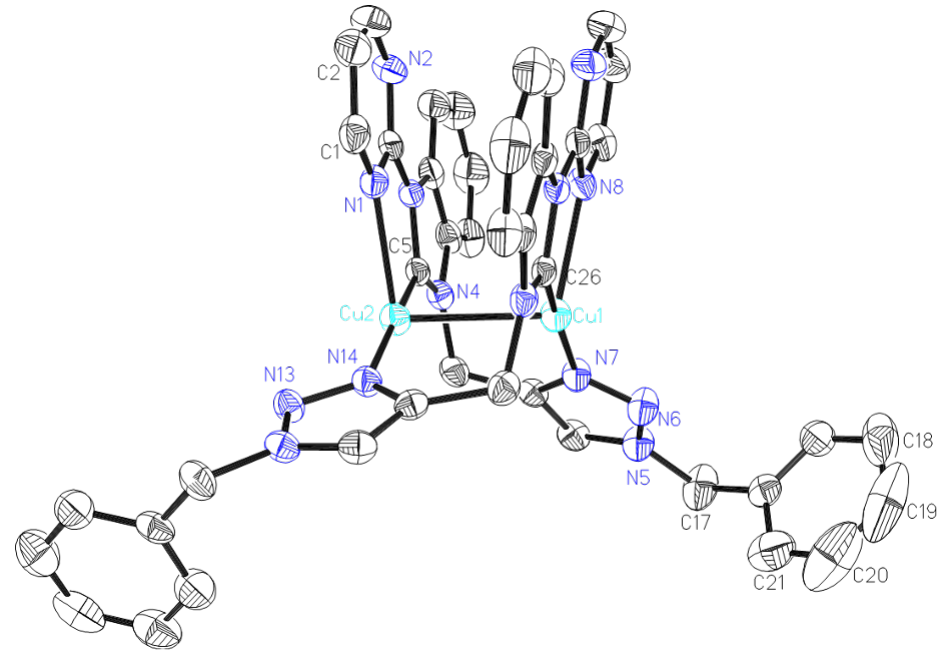
ORTEP the cationic section of [Cu_2_(L_2_)_2_](PF_6_)_2_ (**3**). Thermal ellipsoids are drawn at the 30% probability level. Hydrogen atoms and anions have been removed for clarity. Selected bond distances (Å) and angles (°): Cu2-C5 1.896(3), Cu2-N14 1.911(3), Cu2-N1 2.362(3), Cu2-Cu1 2.7867(7), Cu1-C26 1.898(3), Cu1-N7 1.915(3), Cu1-N8 2.340(3); C5-Cu2-N14 173.37(13), C5-Cu2-N1 77.64(13), N14-Cu2-N1 108.15(12), C5-Cu2-Cu1 69.80(9), N14-Cu2-Cu1 111.70(8), N1-Cu2-Cu1 98.90(7), C26-Cu1-N7 167.08(15), C26-Cu1-N8 78.22(13), N7-Cu1-N8 111.77(13), C26-Cu1-Cu2 73.57(9).

Single crystals of complex **3** suitable for an X-ray diffraction study were grown from acetonitrile solution and diethyl ether. The molecular structure of complex **3** is depicted in Figure 2. Complex **3** crystallizes in the monoclinic space group *C*2/*c*. The Cu(I) complex contains two crystallographically equivalent Cu centers, which are doubly bridged by two L_2_ ligands. The two ligands are arranged in head-to-tail manner. The copper ions are each tri-coordinated by one carbene carbon atom, one nitrogen from pyrimidine, and one nitrogen atom of the triazole rings from two different L_2_ ligands. The Cu–carbene bond distances are 1.896(6) and 1.899(5) Å, which are comparable to the known Cu(I)–NHC complexes [36–39]. The Cu1–Cu2 separation is 2.7867(7) Å, showing a weak metal−metal interaction.

**Figure 3:**
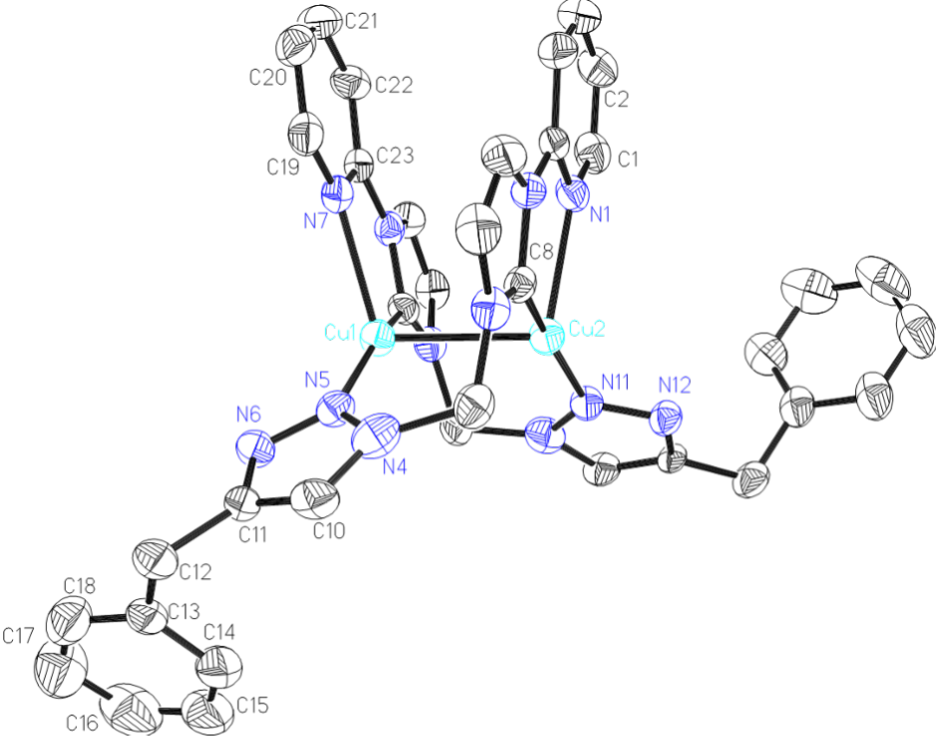
ORTEP drawing of [Cu_2_(L_3_)_2_](PF_6_)_2_ (**4**). Thermal ellipsoids are drawn at the 30% probability level. Hydrogen atoms and anions have been removed for clarity. Selected bond distances (Å) and angles (°): Cu1-C26 1.888(6), Cu1-N5 1.912(5), Cu1-N7 2.289(5), Cu1-Cu2 2.6413(12), Cu2-C8 1.899(5), Cu2-N11 1.922(4), Cu2-N1 2.311(5); C26-Cu1-N5 159.2(2), C26-Cu1-N7 79.0(2), N5-Cu1-N7 116.65(19), C26-Cu1-Cu2 72.49(17), N5-Cu1-Cu2 113.20(15), N7-Cu1-Cu2 105.13(12), C8-Cu2-N11 166.1(2), C8-Cu2-N1 78.6(2), N11-Cu2-N1 110.5(2), C8-Cu2-Cu1 70.45(16), N11-Cu2-Cu1-116.14(15), N1- Cu2-Cu1 102.03(13).

The molecular structure of complex **4** is depicted in Figure 3. Complex **4** consists of the cation unit [Cu_2_(L_3_)_2_]^2+^ and two hexafluorophosphate anions. Complex **4** crystallizes in the triclinic space group *P*-1. The two ligands are also arranged in head-to-tail manner. Each copper ion is three-coordinate in a trigonal planar ligand environment of two nitrogen atoms and one NHC carbon center. The Cu–carbene bond distances are 1.888(6) and 1.899(5) Å which are similar with reported copper-carbene complexes (1.85–2.18 Å) [40]. The Cu1–Cu2 separation is 2.6413(12) Å is shorter than in complex **3**, and slightly higher than reported Cu–Cu separations (2.4907 to 2.5150 Å) of the triangular Cu(I)–NHC clusters [33], showing a weak metal–metal interaction.

**Figure 4:**
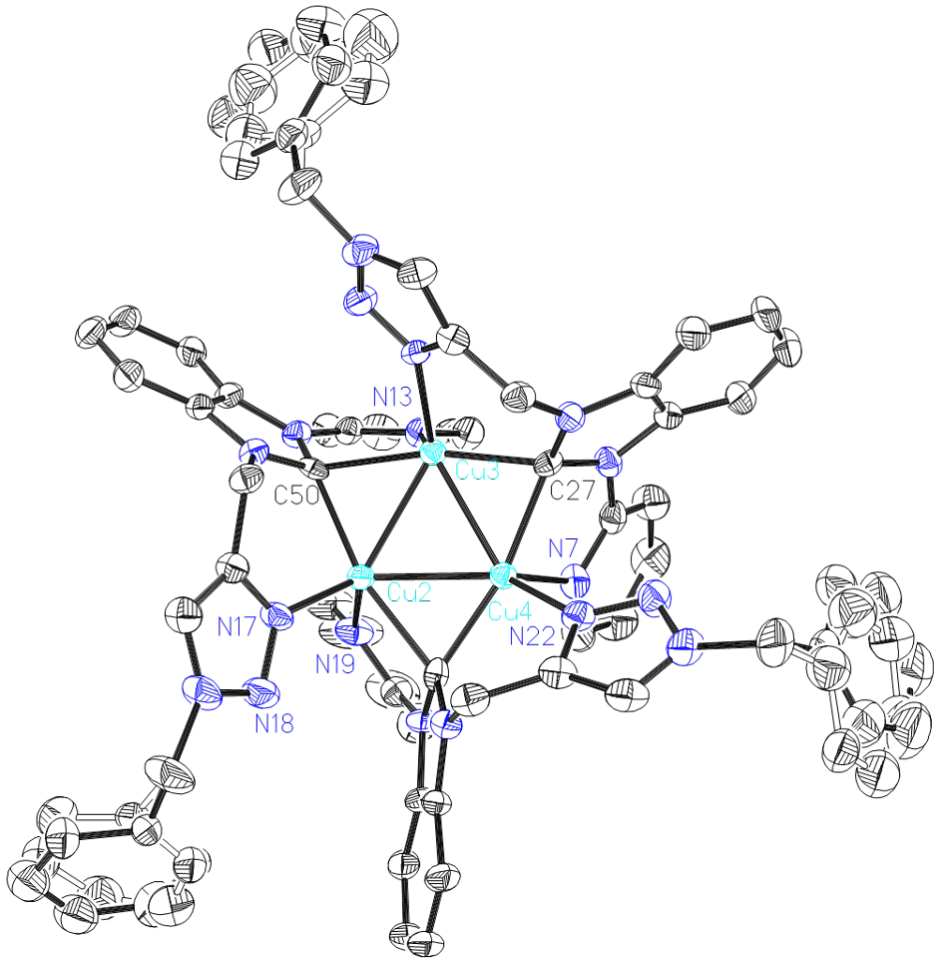
ORTEP drawing of [Cu_3_(L_4_)_3_](PF_6_)_3_ (**5**). Thermal ellipsoids are drawn at the 30% probability level. Hydrogen atoms and anions have been removed for clarity. Selected bond distances (Å) and angles (°): Cu2-N17 2.015(5), Cu2-C50 2.074(6), Cu2-N19 2.107(6), Cu2-C72 2.142(6), Cu2-Cu4 2.4899(11), Cu2-Cu3 2.4928(11), Cu3-N11 2.038(5), Cu3-C27 2.069(6), Cu3-C50 2.113(6), Cu3-N13 2.133(5), Cu3-Cu4 2.4833(10), Cu4-N22 2.043(5), Cu4-C72 2.044(7), Cu4-C27 2.071(6), Cu4-N7 2.086(6); N17-Cu2-C50 94.8(2), N17-Cu2-N19 112.1(2), C50-Cu2-N19 106.8(2), N17-Cu2-C72 93.5(2), C50- Cu2-C72 165.4(2), N19-Cu2-C72 81.0(2), N17-Cu2-Cu4 125.46(18), C50-Cu2-Cu4 113.86(16), N19-Cu2-Cu4 102.90(19), C72-Cu2-Cu4 51.72(17), N17-Cu2-Cu3 129.13(16), C50-Cu2-Cu3 54.18(16), N19-Cu2-Cu3 115.04(17), C72-Cu2-Cu3 111.50(17), Cu4-Cu2-Cu3 59.79(3), N11-Cu3-C27 94.1(2), N11-Cu3-C50 90.9(2), C27-Cu3-C50 163.7(2), N11-Cu3-N13 112.2(2), C27-Cu3-N13 112.1(2), C50-Cu3-N13 80.1(2), N11-Cu3-Cu4 131.09(16), C27-Cu3-Cu4 53.18(17), C50-Cu3-Cu4 112.70(16), N13-Cu3-Cu4 113.70(15), N11-Cu3-Cu2 128.00(15), C27-Cu3-Cu2 112.99(17), C50-Cu3-Cu2 52.76(16), N13-Cu3-Cu2 97.90(16), Cu4-Cu3-Cu2 60.05(3), N22-Cu4-C72 94.4(2), N22-Cu4-C27 92.5(2), C72-Cu4-C27 167.8(2), N22 -Cu4-N7 99.9(2), C72-Cu4-N7 107.5(2), C27-Cu4-N7 81.1(2), N22-Cu4-Cu3 134.20(17), C72-Cu4-Cu3 115.49(17), C27-Cu4-Cu3 53.10(17), N7-Cu4-Cu3 102.67(18), N22-Cu4-Cu2 134.48(17), C72-Cu4-Cu2 55.33(17), C27-Cu4-Cu2 113.02(17), N7-Cu4 -Cu2 120.11(16), Cu3- Cu4- Cu2 60.16(3).

Complex **5** was also characterized via X-ray diffraction. It's structure is shown in Figure 4. Complex **5** consists of two independent molecules in the unit cell. Here, only one molecule was given in Figure 4. The molecule structure consists of a triangular Cu_3_ core bridged by three NHCs ligands. Each NHC forms the 3c-2e bond with two Cu(I) ions with almost equal bond distances (average 2.085 Å), longer than normal Cu–NHC bonds and reported triangular Cu_3_ complexes [33,41]. The Cu_3_ cores of complex **5** possess nearly equilateral angles close to 60°, whereas in complex **6**, the core is crystallographically restrained to an equilateral triangle. The Cu–Cu distances are around 2.4887 Å and are shorten than that of complexe **6,** which may be attributed to more rigid ligand.

**Figure 5:**
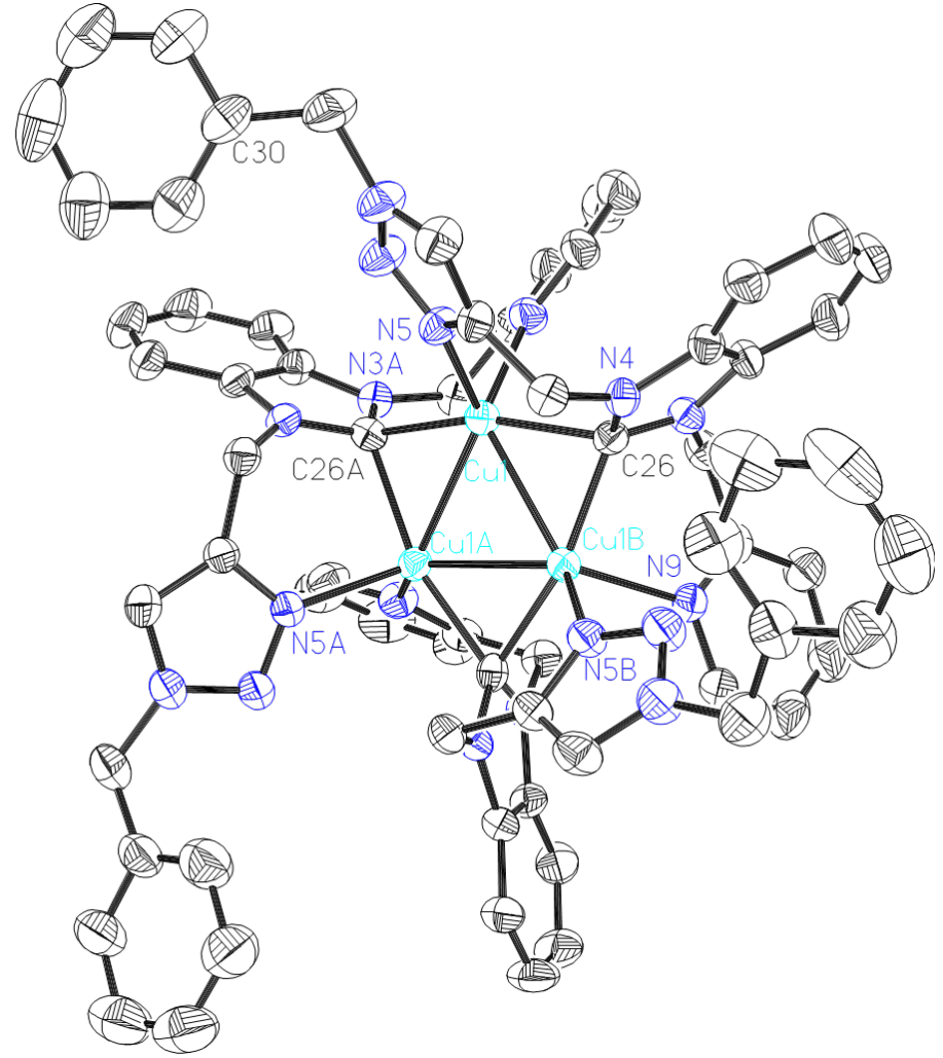
ORTEP drawing of [Cu_3_(L_5_)_3_](PF_6_)_3_ (**6**). Thermal ellipsoids are drawn at the 30% probability level. Hydrogen atoms and anions have been removed for clarity. Selected bond distances (Å) and angles (°): Cu1-C26 2.092(6), Cu1-N5 2.092(5) , Cu1-C26A 2.024(6), Cu1- N9A 2. 152(5), Cu1-Cu1A 2.5141(11), Cu1-Cu1B 2.5141(11); C26-Cu1-N5 101.8(2), C26-Cu1-C26A 163.7(2), N5-Cu1-C26 92.5(2), C26A-Cu1-N9 92.0(2), Cu1-Cu1A-Cu1B 60.0. Symmetry transformations used to generate equivalent atoms: 1−x, 1−y, −z.

Complex **6** has also been characterized by single crystal X-ray diffraction (Figure 5). Complex **6** crystallizes in the hexagonal space group *R*3*c*, which is different to the reported trinuclear copper(I) complex containing the symmetric 1,3-bis(2-pyridinylmethyl)benzimidazolylidene ligand (monoclinic, *P*21/*c*) [33] and to the trinuclear copper(I) complex containing a symmetric 1,3-bis(triazole)benzimidazolylidene ligand (monoclinic, *C*2/*c*) [38]. Three copper atoms are bridged by three N_pyridine_CN_triazole_ NHC ligands forming a Cu_3_ ring with three Cu–Cu–Cu angles of 60.0. The geometry of the copper center can be described as distorted trigonal planar. Each copper ion is coordinated by one pyridine, one triazole, and two benzimidazolylidene ligands displaying a distorted tetrahedral geometry. The Cu–Cu distance is around 2.5145(12) Å showing a weak metal–metal interaction, which is similar with the reported triangle Cu(I) complexes and is shorter than in complexes **3** and **4**. The Cu–N and Cu–C bond distances fall in the range of 2.092(5)–2.152(5) Å and 2.024(6)–2.092(6) Å, respectively, which are slightly longer than in dinuclear complexes **3** and **4**. Benzimidazolylidene acts as a bridging ligand in a *u*2 mode and bonded equally to two Cu(I) ions, which is only observed in a few silver(I) and copper(I) complexes.

### Catalytic application in CuAAC reactions

**Figure 6:**
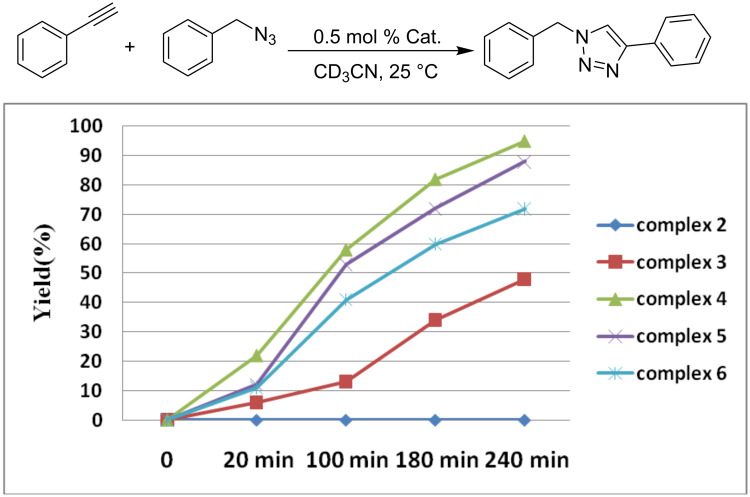
Yield vs reaction time of different copper complex. The reaction was carried out in acetonitrile-*d_3_* at 25 °C using 0.5 mol % copper complex, yields were determined by ^1^H NMR spectra, hexamethylbenzene was used as internal standard.

Inspired by the catalytic activity of Cu(I) species supported by NHC ligand in Cu-catalyzed azide–alkyne cycloaddition (CuAAC) reaction under mild conditions, copper complexes **2**–**6** were investigated in the CuAAC reaction of azide and phenylacetylene. Firstly, we compared the catalytic activity of different complexes with a complex loading of 0.5 mol %. The reactions were monitored by ^1^H NMR analysis at different time points within 4 h (Figure 6). As seen in Figure 6, the yield increased with the extension of reaction time. The results showed that complex **4** displays the best activities for the CuAAC reaction of benzyl azide and phenylacetylene giving a conversion of 95%. To further examine the catalytic efficiency of complex **4**, a variation of the catalyst loading from 0.1 to 0.25 to 0.5 mol % within 5 h was performed to give the expected product in yields of 17%, 48%, and 100%. As expected, the coupling reaction with low catalyst loading results in incomplete conversion.

Subsequently the catalytic activity of different solvents was tested at a Cu loading of 0.5 mol % (Table 1). Moderate catalytic activities were obtained for DMSO or without solvent. When CH_3_CN was used, the reaction gave an excellent yield (Table 1, entry 4). However, only a moderate yield was obtained when a CH_3_CN/H_2_O solvent mixture was used (Table 1, entry 6). Thus, CH_3_CN was selected as the optimal solvent.

**Table 1:** CuAAC reaction with different solvents^a^.

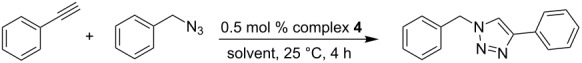			

entry	solvent	cat.	yield %^b^

1	neat	**4**	50
2	H_2_O	**4**	22
3	DMSO	**4**	53
4	CH_3_CN	**4**	95
5	*t*-BuOH/H_2_O (1:1)	**4**	trace
6	CH_3_CN/H_2_O (1:1)	**4**	59

^a^Reaction carried out using 0.5 mol % of complex **4** with different solvents. ^b^Yields were determined by ^1^H NMR spectra and are reported after 4 h, hexamethylbenzene was used as internal standard.

Having optimized the reaction conditions, we extended the CuAAC reaction to other azides and alkynes at room temperature in CH_3_CN. As shown in Table 2 (entries 1–5), (azidomethyl)benzene, azidobenzene, (2-azidoethyl)benzene, and 2-(azidomethyl)pyridine could react with phenylacetylene in more than 83% yield (Table 2, entries 1–4). What is more, methyl 1-benzyl-1*H*-1,2,3-triazole-4-carboxylate could be afforded in 85% yield via reacting methyl propiolate with (azidomethyl)benzene. This promising catalytic behavior of complex **4** prompted us to extend our studies toward a one-pot synthesis of 1,2,3-triazoles from alkyl halides, sodium azide, and alkynes. The three-component version has already been successfully performed and described in previous work [20]. As displayed in Table 2, the reactions proceeded smoothly to completion, and the products were isolated in good to excellent yields (83–95%).

**Table 2:** CuAAC Reaction using complex **4** as catalyst.

entry	substrate	substrate	product	isolated yield %

1^a^	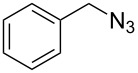	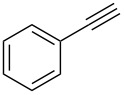	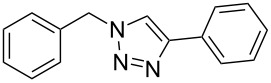	94 [42]
2^a^	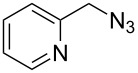	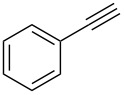	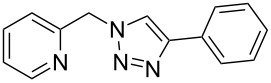	95 [43]
3^a^	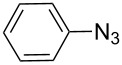	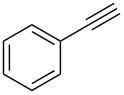	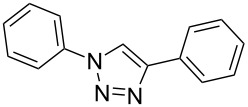	95 [44]
4^a^	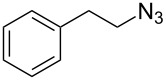	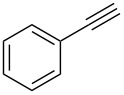	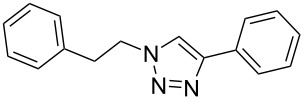	83 [42]
5^a^	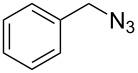	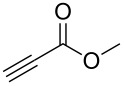	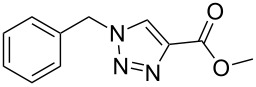	85 [44]
6^b^	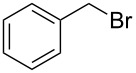	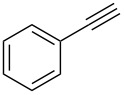	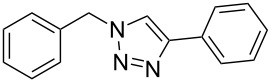	90 [42]
7^b^	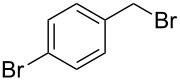	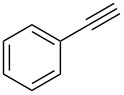	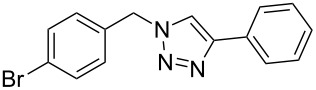	84 [45]
8^b^	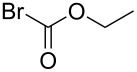	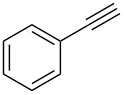	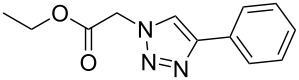	85 [44]

^a^Reaction conditions: azide 0.5 mmol, ethynylbenzene 0.6 mmol, catalyst 0.5 mol %, CH_3_CN 3 mL, rt, 6 h. ^b^Reaction conditions: alkyl halide 0.5 mmol, NaN_3_ 0.6 mmol, ethynylbenzene 0.6 mmol, catalyst 0.5 mol %, CH_3_CN/H_2_O 1:1 (3 mL), rt, 16 h.

## Conclusion

In summary, a series of di-, and trinuclear copper(I) complexes (**3**–**6**) stablized by 1,2,3-triazole-tethered N-heterocyclic carbene ligands have been prepared via simple reactions of imidazolium salts with copper powder in good yields. These complexes have been fully characterized by NMR, elemental analysis (EA) and X-ray crystallography. Fine adjustment of the structure of the ligand can lead to different structures. All the Cu–NHC complexes showed high catalyst activity in CuAAC reactions at room temperature. Among these complexes, complex **4** is the most efficient catalyst in an air atmosphere at room temperature.

## Experimental

All the chemicals were obtained from commercial suppliers and were used without further purification. Elemental analyses were performed on a Flash EA1112 instrument. ^1^H and ^13^C NMR spectra were recorded on a Bruker Avance-400 (400 MHz) spectrometer or a Varian 600 MHz NMR spectrometer. Chemical shifts (δ) are expressed in ppm downfield to TMS at δ = 0 ppm and coupling constants (*J*) are expressed in Hz.

**Synthesis of 3-((1-benzyl-1*H*-1,2,3-triazol-4-yl)methyl)-1-(pyrimidin-2-yl)-1*H*-benzo[*d*]imidazol-3-ium hexafluorophosphate [(HL_2_)PF_6_] (1b):** Analogously as described in a published work [27], (azidomethyl)benzene (160 mg, 1.2 mmol), copper sulfate pentahydrate (12.5 mg, 0.05 mmol), sodium ascorbate (20 mg, 0.1 mmol), and 3-(prop-2-yn-1-yl)-1-(pyrimidin-2-yl)-1*H*-benzo[*d*]imidazol-3-ium bromide (314 mg, 1 mmol) were added to a Schlenk tube containing 2 mL of water and *tert*-butyl alcohol (1:1). After the heterogeneous mixture was stirred vigorously for 24 h at 50 °C, the reaction mixture was diluted with water (20 mL). The obtained yellow solution was dropwise added to the aqueous solution of NH_4_PF_6_. A white precipitate was collected by filtration and dried. Yield: 315 mg, 61%. ^1^H NMR (400 MHz, DMSO-*d*_6_) δ 10.89 (s, 1H), 9.14 (d, *J* = 4.9 Hz, 2H), 8.83 (d, *J* = 8.1 Hz, 1H), 8.39 (s, 1H), 8.17 (d, *J* = 8.0 Hz, 1H), 7.79 (br, 3H), 7.34–7.30 (m, 5H), 6.06 (s, 2H), 5.63 (s, 2H); ^13^C NMR (101 MHz, DMSO-*d*_6_) δ 160.34, 159.78, 143.41, 140.45, 136.12, 132.14, 129.62, 129.26, 128.69, 128.49, 127.79, 125.52, 53.47, 42.95.

**Synthesis of 3-((1-benzyl-1*H*-1,2,3-triazol-4-yl)methyl)-1-(pyridin-2-yl)-1*H*-imidazol-3-ium hexafluorophosphate [(HL_3_)PF_6_] (1c):** Similarly as described in a previous procedure [27], a mixture of (azidomethyl)benzene (160 mg, 1.2 mmol), copper sulfate pentahydrate (12.5 mg, 0.05 mmol) and sodium ascorbate (20 mg, 0.1 mmol), 3-(prop-2-ynyl)-1-(pyridin-2-yl)-1*H*-imidazol-3-ium bromide (265 mg, 1 mmol) was added to 2 mL of water and *tert*-butyl alcohol (1:1). The heterogeneous mixture was stirred vigorously for 24 h at 50 °C. The reaction mixture was diluted with water (20 mL), and the yellow solution was dropwise added to the aqueous solution of NH_4_PF_6_. A white precipitate was collected by filtration and dried. Yield: 415 mg, 90%. ^1^H NMR (400 MHz, DMSO-*d*_6_) δ 10.19 (s, 1H), 8.69–8.63 (m, 1H), 8.54 (t, *J* = 1.9 Hz, 1H), 8.35 (d, *J* = 2.4 Hz, 1H), 8.31–8.17 (m, 1H), 8.03 (q, *J* = 4.3, 3.5 Hz, 2H), 7.70–7.61 (m, 1H), 7.36 (qd, *J* = 7.0, 6.5, 2.5 Hz, 5H), 5.65 (dd, *J* = 5.1, 2.4 Hz, 4H), 3.39 (s, 1H); ^13^C NMR (101 MHz, DMSO-*d*_6_) δ 149.69, 146.78, 141.06, 141.02, 135.68, 129.29, 128.76, 128.55, 125.77, 125.23, 124.14, 120.07, 114.75, 53.49, 44.85.

**Synthesis of 3-((1-benzyl-1*H*-1,2,3-triazol-4-yl)methyl)-1-(pyridin-2-yl)-1*H*-benzo[*d*]imidazol-3-ium hexafluorophosphate [(HL_4_)PF_6_] (1d):** Similarly as described in a previous procedure [27], the imidazolium salt was prepared similarly as for [(HL_3_)PF_6_] from (azidomethyl)benzene (160 mg, 1.2 mmol), copper sulfate pentahydrate (12.5 mg, 0.05 mmol), sodium ascorbate (20 mg, 0.1 mmol), and 3-(prop-2-yn-1-yl)-1-(pyridin-2-yl)-1*H*-benzo[d]imidazol-3-ium bromide (314 mg, 1 mmol). Yield: 317 mg, 62%. ^1^H NMR (400 MHz, DMSO-*d*_6_) δ 10.60 (s, 1H), 8.75 (d, *J* = 4.7 Hz, 1H), 8.46–8.31 (m, 2H), 8.25 (t, *J* = 8.0 Hz, 1H), 8.19–8.10 (m, 1H), 8.02 (d, *J* = 8.2 Hz, 1H), 7.79–7.65 (m, 3H), 7.31 (dt, *J* = 21.5, 7.4 Hz, 7H), 5.95 (s, 2H), 5.59 (d, *J* = 5.1 Hz, 2H); ^13^C NMR (101 MHz, DMSO-*d*_6_) δ 149.97, 147.61, 143.07, 141.07, 140.46, 136.13, 131.72, 130.09, 129.27, 128.74, 128.50, 128.30, 127.73, 125.73, 125.50, 117.68, 116.38, 114.80, 53.42, 42.83.

**Synthesis of 3-((1-benzyl-1*H*-1,2,3-triazol-4-yl)methyl)-1-(pyridin-2-ylmethyl)-1*H*-benzo[*d*]imidazol-3-ium hexafluorophosphate [(HL_5_)PF_6_] (1e):** Similarly as described in previous procedure [27], the imidazolium salt was prepared similarly as for [(HL_3_)PF_6_] from (azidomethyl)benzene (160 mg, 1.2 mmol), and 3-(prop-2-yn-1-yl)-1-(pyridin-2-ylmethyl)-1*H*-benzo[*d*]imidazol-3-ium bromide (328 g, 1 mmol). Yield: 390 mg, 74%.^1^H NMR (400 MHz, DMSO-*d*_6_) δ 10.04 (s, 1H, NCHN), 8.46 (d, *J* = 4.8 Hz, 1H, 2-Py), 8.39 (s, 1H, triazole), 8.10 (d, *J* = 8.0 Hz, 1H, 4-Py), 7.94–7.90 (m, 3H), 7.73–7.59 (m, 3H), 7.46–7.27 (m, 6H, phenyl+benzene), 5.95 (s, 4H, CH_2_), 5.63 (s, 2H, CH_2_);^13^C NMR (101 MHz, DMSO-*d*_6_) δ 153.34, 150.04, 143.67, 140.07, 138.02, 136.14, 131.80, 131.29, 129.27, 128.74, 128.48, 127.33, 127.16, 125.33, 124.19, 123.20, 114.48, 114.41, 53.5, 51.4, 42.3.

**General procedure for the preparation of Cu(I)–NHC complexes and Cu(II) complex:** Analogously as described in [39], all the copper complexes were prepared by the following route: imidazolium salt (0.2 mmol) and an excess of copper powder (64 mg, 1.0 mmol) were placed in 3 mL of MeCN to form a heterogeneous mixture solution. After the mixture was stirred at 50 °C for 10 h under air, the solution was filtered through Celite. Single crystals suitable for X-ray diffraction analysis were grown from acetonitrile solution and diethyl ether.

**Synthesis of [Cu-((1-benzyl-1*H*-1,2,3-triazol-4-yl)methyl)-3-(pyrimidin-2-yl)-1,3-dihydro-2*H*-imidazol-2-one)_2_](PF_6_)_2 _(2):** This complex was synthesized by the reaction of [H(L_1_)](P_F6_) (**1a**; 93 mg, 0.2 mmol) with copper powder (64 mg, 1.0 mmol) at 50 °C for 10 h. Yield: 79 mg (75%), light green crystals. Anal. calcd for C_34_H_30_CuF_12_N_14_O_2_P_2_. 0.5 CH_3_CN: C, 40.39; H, 3.05; N, 19.52; found: C, 40.73; H, 2.95; N, 19.15.

**Synthesis of [Cu_2_(L_2_)_2_](PF_6_)_2_ (3):** This complex was synthesized by the reaction of [HL_2_](PF_6_) (**1b**; 102 mg, 0.2 mmol) with copper powder (64 mg, 1.0 mmol) at 50 ^o^C for 10 h. Yield: 66 mg (57%), red crystals. ^1^H NMR (600 MHz, acetonitrile-*d_3_*) δ 8.75 (d, *J* = 8.1 Hz, 2H, benzimidazole), 8.70 (d, *J* = 4.9 Hz, 4H, pyrimidine), 7.88 (s, 2H, triazole), 7.79 (d, *J* = 7.8 Hz, 2H, benzimidazole), 7.59–7.54 (m, 2H, benzimidazole), 7.52 (t, *J* = 7.2 Hz, 2H, benzimidazole), 7.36 (t, *J* = 4.9 Hz, 2H, pyrimidine), 7.31–7.29 (m, 6H, phenyl), 7.19–7.18 (m, 4H, phenyl), 5.61 (s, 4H, -CH_2_-), 5.38 (s, 4H, -CH_2_-); ^13^C NMR (151 MHz, acetonitrile-*d_3_*) δ 191.23 (Cu-C), 158.73, 157.33 142.39, 136.17 135.75, 132.61, 129.94, 129.67, 129.17, 126.34, 126.19, 125.58, 120.66, 116.95, 112.75, 54.12, 43.41; Anal. calcd for C_42_H_34_Cu_2_F_12_N_14_P_2_: C, 43.80; H, 2.98; N, 17.02; found: C, 43.51; H, 2.85; N, 16.95.

**Synthesis of [Cu_2_(L_3_)_2_](PF_6_)_2 _(4):** The compound was prepared similarly as for complex **3** from [HL_3_](PF_6_) (90 mg, 0.20 mmol) with copper powder (64 mg, 1.0 mmol) at 50 °C for 10 h, orange yellow solid. Yield: 71 mg, 68%. ^1^H NMR (600 MHz, acetonitrile-*d*_3_) δ 7.96 (s, 1H, triazole), 7.90 (br, 1H, 2-py), 7.83 (br, 1H, imidazole), 7.76 (s, 1H, 4-py), 7.57 (br, 1H, imidazole), 7.41 (s, 1H, 5-py), 7.37 (d, *J* = 7.8 Hz, 3H, phenyl), 7.30–7.22 (m, 3H, phenyl + 3-py), 5.47 (s, 2H), 5.38 (s, 2H); ^13^C NMR (151 MHz, acetonitrile-*d*_3_) δ 181.20 (Cu-C), 149.68, 147.26, 140.72, 138.41, 134.71, 129.02, 128.89, 128.80, 128.28, 128.04, 123.96, 123.48, 112.13, 54.36, 45.69; Anal. calcd for C_36_H_32_Cu_2_F_12_N_12_P_2_: C, 41.19; H, 3.07; N, 16.01; found: C, 41.25; H, 3.31; N, 15.46.

**Synthesis of [Cu_3_(L_4_)_3_](PF_6_)_3 _(5):** The compound was prepared similarly as for complex **3** from [HL4](PF_6_) (106 mg, 0.20 mmol) with copper powder (64 mg, 1.0 mmol) at 50 °C for 10 h, light yellow solid. Yield: 93 mg, 52%. ^1^H NMR (600 MHz, acetone-*d*_6_) δ 8.77 (d, *J* = 8.1 Hz, 1H, 2-py), 8.46 (td, *J* = 8.0, 1.8 Hz, 1H, 4-py), 8.33 (s, 1H, triazole), 8.23–8.20 (m, 1H), 7.90 (dd, *J* = 5.1, 1.5 Hz, 1H, benzimidazole), 7.84–7.81 (m, 1H, benzimidazole), 7.68–7.60 (m, 2H, benzimidazole), 7.44 (dd, *J* = 7.6, 5.0 Hz, 1H, 5-py), 7.33–7.31 (m, 3H, phenyl), 6.94–6.92 (m, 2H, phenyl), 5.84 (d, *J* = 15.8 Hz, 1H), 5.37 (d, *J* = 15.8 Hz, 1H), 5.18 (s, 2H); ^13^C NMR (151 MHz, DMSO-*d*_6_) δ 178.99 (Cu-C), 149.61, 148.44, 142.25, 140.55, 136.19, 134.91, 133.34, 129.31, 128.93, 128.09, 126.05, 125.82, 124.48, 124.27, 119.09, 118.51 (CH_3_CN), 112.74, 111.59, 54.13, 41.17, 1.56(CH_3_CN); Anal. calcd for C_132_H_108_Cu_6_F_36_N_36_P_6_. 3CH_3_CN: C, 46.39; H, 3.30; N, 15.29; found: C, 45.87; H, 3.40; N, 15.30.

**Synthesis of [Cu_3_(L_5_)_3_](PF_6_)_3 _(6):** The compound was prepared similarly as for complex **3** from [HL5](PF_6_) (106 mg, 0.20 mmol) with copper powder (64 mg, 1.0 mmol) at 50 °C for 10 h, light yellow solid. Yield: 106 mg, 90%. ^1^H NMR (600 MHz, nitromethane-*d*_3_) δ 8.13 (s, 1H, triazole), 7.92 (td, *J* = 7.8, 1.8 Hz, 1H, pyridine), 7.77 (d, *J* = 7.8 Hz, 1H, pyridine), 7.59 (d, *J* = 7.8, 1H, pyridine), 7.61–7.36 (m, 6H, phenyl + benzimidazole), 7.14–7.08 (m, 2H, benzimidazole), 6.95 (ddd, *J* = 7.5, 5.2, 1.1 Hz, 1H, pyridine), 6.49–6.45 (d, *J* = 4.8 Hz,1H, benzimidazole), 5.40 (d, *J* = 15.0 Hz, 1H, -CH_2_-), 5.30 (d, *J* = 15.0 Hz, 1H, -CH_2_-), 5.27 (d, *J* = 15.0 Hz, 1H, -CH_2_-), 5.26 (d, *J* = 15.6 Hz, 1H, -CH_2_-), 5.21 (d, *J* = 15.0 Hz, 1H, -CH_2_-), 4.98 (d, *J* = 15.6 Hz, 1H, -CH_2_-); ^13^C NMR (150 MHz, nitromethane-*d*_3_) 177.57 (Cu-C), 151.99, 148.85, 141.78, 139.93, 135.15, 134.68, 133.85, 129.16, 128.98, 128.81, 128.12, 125.04, 124.62, 124.35, 123.79, 110.58, 110.26, 54.49, 51.29, 40.72; Anal. calcd for C_69_H_60_Cu_3_F_18_N_18_P_3_: C, 46.90; H, 3.42; Cu, 10.79; N, 14.27; found: C, 46.35; H, 3.31; N, 13.95.

**General procedure for the copper-catalyzed CuAAC reaction:** Analogously as described in [31], in a 10 mL Schlenk tube, azide (0.5 mmol), alkyne (0.6 mmol), and 0.5 mol % copper complex were dissolved in 3.0 mL of CH_3_CN. After the mixture was stirred at rt under air for a desired time, the reaction was stopped by the addition of H_2_O (2 mL) to the resultant mixture. Then the mixture was extracted with CH_2_Cl_2_. The organic layer was separated from the aqueous phase. After the organic phase was dried over MgSO_4_, the solution was filtered and concentrated under vacuum. The residue was purified by flash chromatography (silica gel, petroleum ether/ethyl acetate, 3:1) to give the desired product

### X-ray diffraction analysis

Analogously as described in [27], single-crystal X-ray diffraction data were collected at 298(2) K on a Siemens Smart/CCD area-detector or Oxford Diffraction Gemini A Ultra diffractometer with a Mo Kα radiation (λ = 0.71073 Å) by using an ω-2θ scan mode. Unit-cell dimensions were obtained with least-squares refinement. Data collection and reduction were performed using the SMART and SAINT software [46]. The structures were solved by direct methods, and the non-hydrogen atoms were subjected to anisotropic refinement by full-matrix least squares on *F*_2_ using the SHELXTXL package [47]. Hydrogen atom positions for all of the structures were calculated and allowed to ride on their respective C atoms with the C–H distances of 0.93–0.97 Å and *U*_iso_(H) = 1.2 − 1.5*U*_eq_(C). Disordered solvent molecules that could not be modeled successfully were removed with SQUEEZE [48]. Further details of the structural analysis are summarized in Table 3.

**Table 3:** Crystallographic data for complexes **2**–**6**.

	**2**	**3**	**4**	**5**	**6**

CCDC number	1424013	1424014	1424015	1424016	1424017
formula	C_70_H_63_Cu_2_F_24_N_29_O_4_P_4_	C_42_H_34_Cu_2_F_12_N_14_P_2_	C_36_H_32_Cu_2_F_12_N_12_P_2_	C_278_H_237_Cu_12_F_72_N_79_P_12_	C_69_H_60_Cu_3_F_18_N_18_P_3_
Fw.	2081.47	1161.93	1049.76	7186.59	1766.88
crystal system	orthorhombic	monoclinic	triclinic	hexagonal	hexagonal
space group	*Pnna*	*C*2/*c*	*P-1*	*R*3*c*	*R*3*c*
*a*/Å	14.0847(15)	34.298(3)	12.9626(10)	28.178(3)	21.4692(13)
*b*/Å	14.5426(16)	13.2602(12)	13.0382(9)	28.178(3)	21.4692(13)
*c*/Å	22.599(2)	26.605(4)	13.1663(10)	16.3997(19)	71.858(9)
β/deg	90.00	129.8310(10)	80.661(6)	90.00	90.00
V/Å^3^	4629.0(9)	9291.9(19)	2111.7(3)	45655(10)	28684(4)
Z	2	8	2	6	12
*D*/g cm^−3^	1.493	1.661	1.651	1.568	1.228
Reflns collected	18121	10632	7571	21009	5624
ind reflns, Rint	12510, 0.0545	7828, 0.0250	4679, 0.0467	15722, 0.0869	3716, 0.0650
goodness-of-fit on F2	1.063	1.018	1.023	1.031	1.157
R1, wR2 [I > 2σ(I)]	0.0869, 0.2496	0.0562, 0.1652	0.0696, 0.1774	0.0622, 0.1353	0.0688, 0.2044
R1, wR2 (all data)	0.1338, 0.3001	0.0779, 0.1858	0.1122, 0.2172	0.0917, 0.1543	0.1200, 0.2623

## Supporting Information

File 1 X-ray crystallographic data in cif format CCDC 1424013–1424017.

## References

[1] Díez-González S, Marion N, Nolan S P (2009). Chem Rev.

[2] Poyatos M, Mata J A, Peris E (2009). Chem Rev.

[3] Bernhammer J C, Han Vinh H (2014). Organometallics.

[4] Farrell K, Albrecht M, van Koten G, Gossage R A (2016). Late Transition Metal Complexes with Pincer Ligands that Comprise N-Heterocyclic Carbene Donor Sites. The Privileged Pincer-Metal Platform: Coordination Chemistry & Applications.

[5] Velazquez H D, Verpoort F (2012). Chem Soc Rev.

[6] Gaillard S, Cazin C S J, Nolan S P (2012). Acc Chem Res.

[7] Lazreg F, Nahra F, Cazin C S J (2015). Coord Chem Rev.

[8] Prakasham A P, Ghosh P (2015). Inorg Chim Acta.

[9] Mata J A, Hahn F E, Peris E (2014). Chem Sci.

[10] Fortman G C, Nolan S P (2011). Chem Soc Rev.

[11] Mejuto C, Royo B, Guisado-Barrios G, Peris E (2015). Beilstein J Org Chem.

[12] Schulte to Brinke C, Hahn F E (2015). Dalton Trans.

[13] Ibrahim H, Bala M D (2015). J Organomet Chem.

[14] Chen C, Lu C, Zheng Q, Ni S, Zhang M, Chen W (2015). Beilstein J Org Chem.

[15] Liu B, Liu B, Zhou Y, Chen W (2010). Organometallics.

[16] Guo T, Dechert S, Meyer F (2014). Organometallics.

[17] Zhang X, Xi Z, Liu A, Chen W (2008). Organometallics.

[18] Liu X, Chen W (2012). Organometallics.

[19] Gu S, Liu B, Chen J, Wu H, Chen W (2012). Dalton Trans.

[20] Gu S, Xu D, Chen W (2011). Dalton Trans.

[21] Crowley J, McMorran D, Košmrlj J (2012). “Click-Triazole” Coordination Chemistry: Exploiting 1,4-Disubstituted-1,2,3-Triazoles as Ligands. Click Triazoles.

[22] Sluijter S N, Elsevier C J (2014). Organometallics.

[23] Warsink S, Drost R M, Lutz M, Spek A L, Elsevier C J (2010). Organometallics.

[24] Huang D, Zhao P, Astruc D (2014). Coord Chem Rev.

[25] Zamora M T, Ferguson M J, McDonald R, Cowie M (2012). Organometallics.

[26] Vuong K Q, Timerbulatova M G, Peterson M B, Bhadbhade M, Messerle B A (2013). Dalton Trans.

[27] Gu S, Xu H, Zhang N, Chen W (2010). Chem – Asian J.

[28] Lu C, Gu S, Liu X (2014). Inorg Chem Commun.

[29] Gu S, Xu W, Huang J (2013). Prog Chem.

[30] Gu S, Huang J, Chen W (2013). Chin J Org Chem.

[31] Gu S, Huang J, Liu X, Liu H, Zhou Y, Xu W (2012). Inorg Chem Commun.

[32] Liu B, Zhang Y, Xu D, Chen W (2011). Chem Commun.

[33] Chen C, Qiu H, Chen W (2012). J Organomet Chem.

[34] Lin J C Y, Huang R T W, Lee C S, Bhattacharyya A, Hwang W S, Lin I J B (2009). Chem Rev.

[35] Charra V, de Frémont P, Breuil P-A R, Olivier-Bourbigou H, Braunstein P (2015). J Organomet Chem.

[36] Liu B, Pan S, Liu B, Chen W (2014). Inorg Chem.

[37] Collins L R, Lowe J P, Mahon M F, Poulten R C, Whittlesey M K (2014). Inorg Chem.

[38] Liu B, Ma X, Wu F, Chen W (2015). Dalton Trans.

[39] Liu B, Chen C, Zhang Y, Liu X, Chen W (2013). Organometallics.

[40] Pouy M J, Delp S A, Uddin J, Ramdeen V M, Cochrane N A, Fortman G C, Gunnoe T B, Cundari T R, Sabat M, Myers W H (2012). ACS Catal.

[41] Catalano V J, Munro L B, Strasser C E, Samin A F (2011). Inorg Chem.

[42] Appukkuttan P, Dehaen W, Fokin V V, Van der Eycken E (2004). Org Lett.

[43] Urankar D, Pevec A, Turel I, Košmrlj J (2010). Cryst Growth Des.

[44] Campbell-Verduyn L S, Mirfeizi L, Dierckx R A, Elsinga P H, Feringa B L (2009). Chem Commun.

[45] Chassaing S, Sani Souna Sido A, Alix A, Kumarraja M, Pale P, Sommer J (2008). Chem – Eur J.

[46] (1996). SMART-CCD Software.

[47] (1997). SHELXS-97 and SHELXL-97, Program for X-ray Crystal Structure Refinement.

[48] Spek A L (1998). PLATON, A Multipurpose Crystallographic Tool.

